# Effect of milk fat globules on growth and metabolism in rats fed an unbalanced diet

**DOI:** 10.3389/fnut.2023.1270171

**Published:** 2024-01-11

**Authors:** Nurit Argov-Argaman, Hodaya Altman, Jerome Nicolas Janssen, Seman Daeem, Chen Raz, Ronit Mesilati-Stahy, Svetlana Penn, Efrat Monsonego-Ornan

**Affiliations:** ^1^Department of Animal Sciences, Hebrew University of Jerusalem, Jerusalem, Israel; ^2^School of Nutrition Science, Institute of Biochemistry, Hebrew University of Jerusalem, Jerusalem, Israel

**Keywords:** milk and dairy products, metabolism, lipids, bone, milk fat globule (MFG)

## Abstract

We assessed the effects of supplementing milk fat globules (MFG) on the growth and development of the skeleton in rats fed a Western unbalanced diet (UBD). The UBD is high in sugar and fat, low in protein, fiber, and micronutrients, and negatively impacts health. The MFG—a complex lipid-protein assembly secreted into milk—has a unique structure and composition, which differs significantly from isolated and processed dietary ingredients. Rats consuming the UBD exhibited growth retardation and disrupted bone structural and mechanical parameters; these were improved by supplementation with small MFG. The addition of small MFG increased the efficiency of protein utilization for growth, and improved trabecular and cortical bone parameters. Furthermore, consumption of UBD led to a decreased concentration of saturated fatty acids and increased levels of polyunsaturated fatty acids (PUFA), particularly omega-6 PUFA, in the serum, liver, and adipose tissue. The addition of small MFG restored PUFA concentration and the ratio of omega-6 to omega-3 PUFA in bone marrow and adipose tissue. Finally, large but not small MFG supplementation affected the cecal microbiome in rats. Overall, our results suggest that natural structure MFG supplementation can improve metabolism and bone development in rats fed an UBD, with the effects depending on MFG size. Moreover, the benefits of small MFG to bone development and metabolism were not mediated by the microbiome, as the detrimental effects of an UBD on the microbiome were not mitigated by MFG supplementation.

## Introduction

Consumption of milk at all ages, but especially in infancy and childhood, affect the microbiome, metabolism, and growth. Differences in bone quality have been associated with the consumption of breast milk vs. infant formula during infancy, as well as with the consumption of milk and dairy products in childhood [reviewed by Yackobovitch-Gavan et al. (1)]. The exact mechanism is not clear and contradictory results are often reported. While the source of protein and carbohydrates in infant formula is usually bovine or caprine milk and fermented sugars, plant-derived oil generally provides the fat. The fat fraction in milk differs fundamentally from oil, and effort has therefore been invested in mimicking the composition and structure of milk fat in infant formula (2). Milk fat consists of over 400 different fatty acids (FA), phospholipids, cholesterol, and glycoconjugates, which are secreted in a complex and unique macrostructure termed milk fat globule (MFG) (3) which is profoundly different from plant derived oils. This structure is common to milk of all mammalian species, including human, bovine, caprine, and murine. The MFG consists of a triglyceride core enveloped by a trilayer of proteins and polar lipids termed MFG membrane (MFGM) (3).

MFGM supplementation has been found to ameliorate hyperglycemia and improve glucose metabolism in the liver and skeletal muscle of mice with type 2 diabetes induced by a high-fat diet and streptozotocin (4). It has also been found to reduce weight gain in rats when supplemented to a high-fat diet (5), and to reduce inflammatory markers in obese human subjects (6). Health-promoting effects of MFGM have also been demonstrated specifically in the gut, including alleviation of foodborne infections (7) and decreased rates of gut infection in infants (8–10), as well as improved microbiome, as manifested by reduced gut colonization by a Listeria strain in adult rats (11), and reduced opportunistic pathogens in the gut of pre-weaned mice (12, 13). MFGM supplementation has also been found to protect probiotic bacteria, such as lactobacilli, from digestive processes and bile stress in rats (14).

One of the complexities conferred by the structure of milk fat is the diversity of MFG sizes, ranging from the nanometer scale to over 15 μm with an average size of ~3.6 μm, and the close association between MFG size and composition (15, 16). Specifically, smaller globules have a relatively higher mass ratio of MFGM (16), and may therefore exert bioactivities that differ from those of larger globules. The first physiological role assigned to the size of natural, intact MFG was in modulating *Bacillus subtilis* metabolism, growth, and the ability to form biofilm (17). The role of size has also been demonstrated as lower fat accumulation in mice that received large artificial lipid droplets coated with milk polar lipids instead of a standard formula with oil as the lipid source (18).

The effects of an unbalanced diet (UBD) during childhood on metabolic and growth trajectories have been well documented. The Western UBD (19) is characterized by high fat and low protein, fiber, and micronutrients (20, 21). Its consumption promotes all forms of malnutrition, and is associated with overweight and obesity (22–25), as well as stunting (26–29). The UBD is also associated with dysbiosis—a condition that changes the composition and diversity of the gut microbiome (30), and among others, correlates with the production of proinflammatory cytokines regulating osteogenic cells (31, 32). In rats, it has been shown to influence hormonal somatotrophic (GH/IGF1) axis activity to drive bone growth and bone mass (33–35). In addition, short-chain FA produced by the gut microbiome may directly affect dietary calcium absorption and hence bone mineralization (36). Although the impact of UBD on metabolism and growth was extensively studied, the impact of such diets as a whole on skeletal development are scarce (30, 37, 38).

Taken together, these data suggest that an UBD affects bone quality both directly and indirectly through modulation of the gut microbiome, inflammatory status, calcium absorption, and overall metabolism. The consumption of milk’s structured lipids may alleviate these conditions since it was demonstrated that it can affect microbiome and consequently inflammatory state of the gut. Since the relative abundance of polar lipids is heavily dependent on the MFG size, we aimed to investigate the role played by MFG size in regulating growth, metabolism, and bone development under UBD. We hypothesized that the native macrostructure of MFG plays a role in bone development, and may contribute to bone quality when supplemented to an UBD. In addition, we hypothesized that MFG size has a distinct role in microbiome remodeling and consequently, in bone development. The current study contribute to our understanding of the importance of the macrostructure and food matrix, which have a role in growth development and health at early stages of life, specifically under unbalanced, deficient diet.

## Materials and methods

### Experimental design

**Figure 1:**
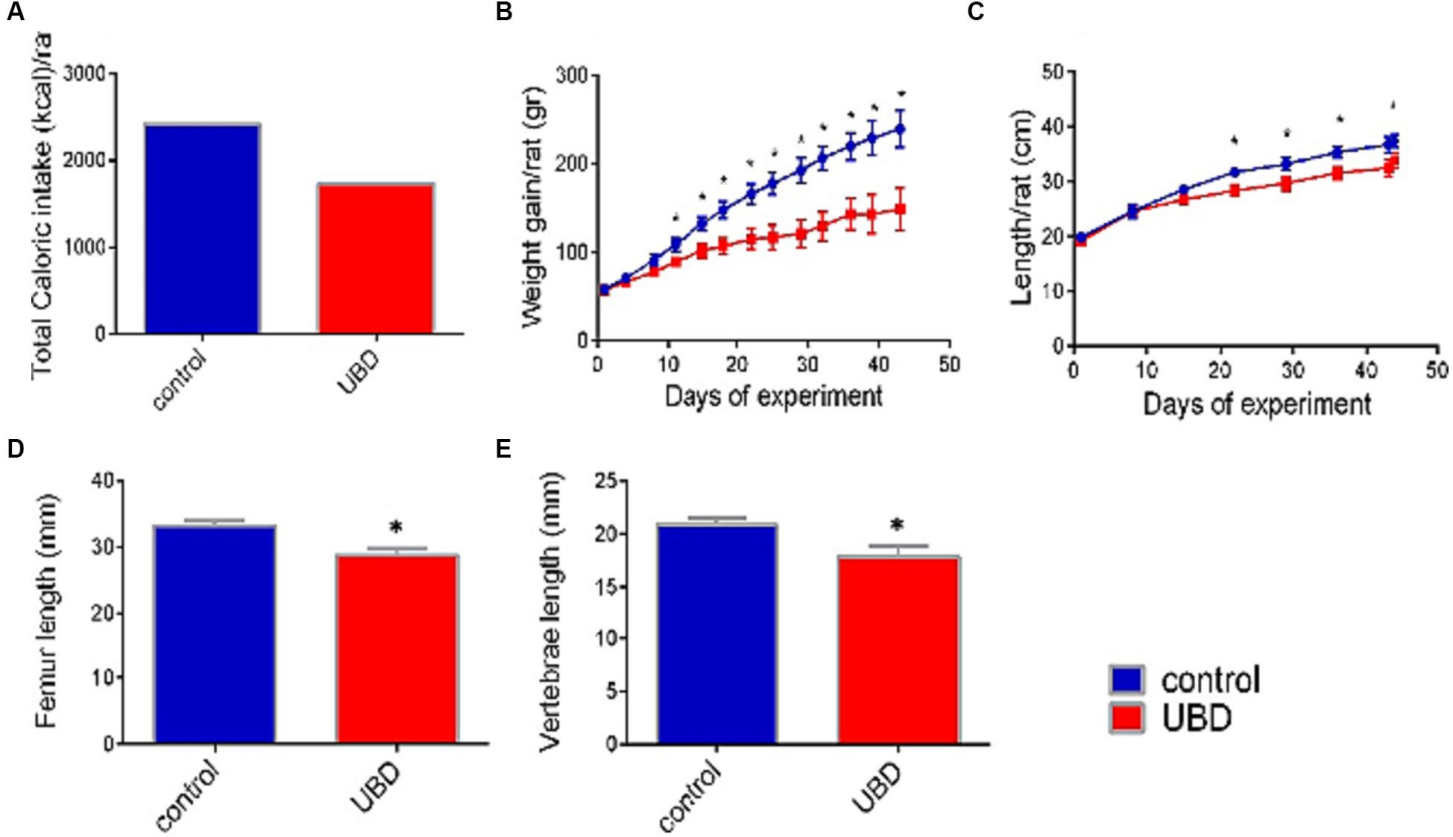
Effect of UBD on rat growth pattern and caloric intake during the experiment. **(A)** Total caloric intake (kcal/rat). **(B)** Weight gain (g/rat). **(C)** Longitudinal growth (cm/rat). **(D)** Femur length (mm). **(E)** Vertebrae length (mm). **(A)** Values are expressed as mean of 2 cages for each diet and calculated for the estimated consumption for rat. **(B–E)** Values are expressed as mean ± SD of *n* = 8 rats/group. Asterisk indicate significant difference (*p* < 0.05) by one-way ANOVA followed by Tukey’s test.

In order to evaluate the influence of UBD on skeletal development, a 6-week long experiment was conducted on Sprague–Dawley (SD) rats after weaning (from 3 to 9 weeks old). This time frame was selected in order to mimic the human growth period up to sexual maturity (39). Female SD rats (*n* = 32, Harlen Laboratories, Rehovot, Israel) were housed in standard environmental conditions, with 12 h light: 12 h dark cycle, a controlled temperature (23°C ± 1°C) and *ad-libitum* access to water and food. After 4 days of adaptation to normal chow diet, we randomly divided the rats into 4 groups (Figure 1). The first, the control, received a normal diet as recommended for growing rats (30). The other three received an UBD with low protein (10% of kcal), low mineral and vitamin (50% of the recommended amount) and high fat (25% of kcal): UBD group (UBD) that served as a reference for growth under nutritional deficiencies; UBD supplemented with small MFG group (UBD + small MFG); and UBD supplemented with large MFG group (UBD + large MFG). Throughout the experiment body mass (g) and food intake (g) were measured twice a week. Additionally, rat’s length (cm) was evaluated once a week.

At the end of the experiment, 9 weeks post-partum, rats were sacrificed, serum samples were stored at −80°C and various organs (intestine, liver, fat tissue, femur, tibia) of the rats were harvested for further analyses (38).

### Diet preparation and composition

The control group received a diet based on the recommendations of the American Institute of Nutrition (AIN)-93G formulated for the growth phase of rodents: 16% caloric from fat, 63.5% caloric from carbohydrate, and 20.5% caloric from protein (40) (Table 1). The other groups received an UBD with caloric composition: 25% fat, 65% carbohydrates and 10% protein (Tables 1, 2) and 50% of vitamin and mineral mix. Due to the higher levels of fat in the UBD, the diets were not isocaloric, UBD contains 3.8 kcal/g and control diet 3.49 kcal/g.

**Table 1:** Macronutrient and mineral composition of the experimental diets.

	Control diet	Unbalanced diet
Ingredient	g/kg diet	g\kg diet
Cornstarch	397	438.7
Casein (≥85% protein)	200	105
Dextrinized cornstarch (90%–94% tetrasaccharides)	132	145.7
Sucrose	100	110
Soybean oil	70	118.8
Fiber	50	54
Mineral mix (AIN-93G-MX)	35	18.9
Vitamin mix (AIN-93-VX)	10	5.4
L-Cystine	3	1.6
Choline bitartrate (41.1% choline)	2.5	1.34
Tert-butylhydroquinone	0.014	0.015

**Table 2:** Caloric composition of the experimental diets.

	Control diet		Deficient diet	
Ingredient	Kcal/kg	% Kcal	Kcal\kg	% Kcal
Cornstarch	1,402	63.5	1559.4	65
Dextrinized cornstarch (90%–94% tetrasaccharides)	465.5		518	
Sucrose	352.7		392	
Casein (≥85% protein)	705	20.5	374	10
L-Cystine	10.6		5.6	
Soybean oil	555.5	16	949.9	25
Total calories	3491.3		3,799	
Kcal\g	3.49		3.799	

Values are expressed as kcal per Kg diet including the addition of water.

Food consumption was collected from 2 cages per experimental group, with 4 rats in each cage. The constellation chosen for the experiment, as required by ethics, does not enable the calculation of significance in food consumption. The other measurements (anthropometric, bone parameters, fat analyses and microbiome) were done on each rat separately (*n* = 8) thus allowed the statistical analyses. Food efficiency was calculated by the added BW of each rat divided by the average caloric intake, protein, fat, or carbohydrate consumption. The measurements of food consumption in each cage were used to calculate these parameters (which varied between the different diets).

### Milk fat globules preparation

Raw milk of a commercial herd containing 300 dairy cows, was separated by conventical milk separator (Beit El, Binyamina) to cream (large MFG) and skim (small MFG). Solid composition was determined by near infra-red spectroscopy (Lactoscan, FOSS). MFG size was determined in both fractions using light scattering (Mastersizer, Malvern, United Kingdom). The average diameter of large MFG was 3.4 μm and the small MFG was 2.4 μm. The concentration of fat was normalized with water to reach the level of 1.48 and 2.2 g/100 mL solution, and 1 mL was administrated daily by oral gavage to experimental animals. The protein and lactose composition was 5.8 and 5.4 g/100 m for the small MFG supplement and 0.46 and 0.33 g/100 mL for the large MFG.

### Bone microarchitecture

Femora were scanned using a Skyscan 1,174 (Skyscan, Bruker, Belgium) X-ray computed micro-tomography device. Images were obtained at 50 kV X-ray tube voltage and 800 μA current, using a 0.25 mm aluminum filter, 4,000 ms exposure time, and 15 μm optical resolution. For each specimen, a series of 900 projection images were obtained (a rotation step of 0.4°, averaging 2 frames, for a total 360° rotation). A stack of 2-D X-ray shadow projections was reconstructed to obtain images using NRecon software (Skyscan, Bruker, Belgium). Next, images were subjected to morphometric analysis using CTAn software (CT Analyser 1.13.5.1, Skyscan, Bruker, Belgium). Morphometric parameters were calculated as suggested by recent guidelines for bone microstructure assessment. To analyze the diaphyseal cortical region, 200 slices, centered at the mid diaphysis, equivalent to 2.764 mm, were chosen. Global grayscale threshold levels for the cortical region were between 71 and 255. For the trabecular region, a total of 150 slices, equivalent to 2.073 mm of the bone, were selected, and adaptive grayscale threshold levels between 58 and 255 were used. Two phantoms with known density (0.25 and 0.75 g/cm3) were scanned under the same conditions of the femora samples allowing to measure the cortical diaphysis BMD (bone mineral density); quantification were carried out using CTAn software (41, 42). The 3rd–5th lumbar vertebrae were scanned and analyzed as well. The spatial resolution was 18 μm and the total rotation was 180°, except that all other parameters were identical to the femoral scans. The region chosen for the analysis was manually selected and consisted of 120 slices of the 5th vertebra starting from the proximal end-plate. Adaptive grayscale threshold levels between 66 and 255 were selected for the analysis of the trabecular vertebral region.

The length of the femora and 3rd–5th segments of the lumbar vertebrae were measured using the Micro-CT device prior to the scans. By using the Amira software (v.6.4, FEI, Hillsboro, OR, United States) reconstructed scans were volume-rendered to visualize the 3D morphology and BMD variation using visualization, of the selected sample from each group.

Three Point Bending for Bone Mechanical Analysis Femora were tested using an Instron mechanical tester (Model 3345). Each bone was placed within a custom-built saline containing testing chamber and on two supports having rounded profiles (2 mm in diameter), so that the supports were in touch with the posterior aspect of the diaphysis. The distance between the stationary supports was set to 10 mm, to ensure that the relatively tubular portion of the mid-diaphysis rests on these supports. A pronged loading device was applied to the anterior surface of the bones, precisely in the middle between the two supports. First, an initial preload of 0.1 N was applied to hold the bone in place; following that, the prong was advanced at a constant rate of 600 μm/min, loaded up to the fracture point, identified by a sudden >20% decrease in load (42).

Force-displacement data were collected by Instron software BlueHill (version 2.0, Instron Corporation, Norwood, Massachusetts, United States) at 10 Hz. The resulting force-displacement curves were used to calculate bone stiffness, bone yield point, load of fracture, maximal load and area under the curve was measured to calculate the total energy to fracture (E to F) (43).

### Histological analysis

Tibial growth plates (GPs) were fixed overnight in 4% paraformaldehyde (Sigma, United States) followed by 2 weeks of decalcification in 0.5 M EDTA pH 7.4. The samples were then dehydrated and transferred into histoclear (Bar-Naor) and subsequently, embedded in paraffin. Transverse tissue sections of 5 μm were prepared with Leica microtome (Agentec, Israel). For H&E histological staining, sections were deparaffinized and rehydrated (44), and stained in hematoxylin solution followed by eosin The sections for all histological analyses were dried and mounted with DPX mounting for histology. The thickness of total GP was measured using the Cell A software (Olympus) with a measuring tool feature at 10 selected locations throughout the GP in 4 different samples at each group. For imaging, the stained sections were viewed by the light microscopy Eclipse E400 Nikon. Images were captured by a high-resolution camera (DP71 Olympus), controlled by Cell A software (Olympus) (45).

### Fatty acids analysis in tissues using gas chromatography

Analysis of FA composition of the MFG, diets, liver, adipose tissue and bone marrow tissue samples were performed by the laboratory of Dr. Nurit Argov-Argaman as described (16). At the day of analysis, food dumpling from the control and the UBD were grinded and 125 mg were taken to analysis. In addition, the liver and serum were slowly thawed to room temperature. 200 mg of liver tissue and 250 μL of serum were taken to analysis. Adipose tissue was collected from the visceral fat. At the day of the analysis, adipose tissue was slowly thawed to room temperature, and 150 mg was taken from the tissue. Bone marrow was isolated from the right tibiae. Tibiae were cut at the end of the distal epiphysis and centrifuged for 1 min in 15,000 g. bone marrow was collected, weighted and stored at −20°C until analysis. Before analysis, all tissues were grinded with tissues grinder.

Gas chromatographic (GC) analysis was performed in a GC (Agilent Technologies, Santa Clara, CA) equipped with a fused-silica capillary column (60 m × 0.25 mm ID, DB-23, Agilent) as previously describe (3). Peaks were identified by comparison with retention times of two external standards: for polyunsaturated FA (PUFA), PUFA-2 (Sigma Aldrich Israel Ltd., Rehovot, Israel), and a FAME C8:0 to C24:0 mix (Supelco, Bellefonte, PA, United States). FA with the same chemical composition were grouped (sum of mol% values) into saturated FA (C8:0-C24:0). MUFA (C16:1n-7, C18:1n-9, C18:1n-7, C20:1n-9, C22:1n-9) and PUFA (C18:2n-6, C18:3n-6, C18:3n-3, C20:4n-6, C20:5n-3, C22:4n-6, C22:6n-3). In addition, FA were grouped into omega-3 (C18:3n-3, C20:5n-3 and C22:6n-3) and omega-6 (C18:2n-6, C18:3n-6, C20:4n-6 and C22:4n-6) FA.

### Microbiome analysis

On the sacrifice day, caecum from of 30 rats (*n* = 7 rats/group for control and UBD + large MFG, and *n* = 8 rats/group for UBD and UBD + small MFG) was collected and stored at −80°C. 0.25 g of defrosted caecum content was extracted using the QIAamp PowerFecal kit (Qiagen, Hilden, Germany) according to the manufacturer’s instructions. The DNA extraction was stored at −80°c until sent in dry ice to the University of Illinois in Chicago for 16S rRNA sequencing of the V4 region using 515F–806R primers. The DADA2 amplicon workflow (v1.24) (46) was used to process forward and reverse reads. Taxonomy was classified using DECIPHER (v2.24) and the RDP (v18) training set. Alpha- and beta diversity were assessed using the phyloseq (v1.40) and vegan (v2.6.4) packages. Phylogentic tree for PCoA weighted UniFrac analysis was calculated with FastTree (v. 2.1.11) using standard parameters. DESeq2 (v1.36.0) was used for differential abundance analysis and results were plotted using pheatmap (v1.0.12), excluding bacteria not classified on genus level. The 16S rRNA datasets are available at NCBI Gene Expression Omnibus (GEO), GEO accession: GSE239878.

### Statistical analysis

All data is expressed as mean ± SD. The significance of differences between groups was determined using JMP 14.0.0 Statistical Discovery Software (SAS Institute 2000) by one-way analysis of variance. Differences between groups were further evaluated by the Tukey–Kramer HSD test, considered significant at *p* < 0.05. Microbiome analysis was conducted under the following conditions: For distribution of the bacterial taxa at the phylum level only amplicon sequence variants (ASVs) passing the minimum frequency of 1% were included. Alpha diversity was calculated using the Shannon index and a pairwise Wilcoxon rank sum test with false discovery rate (FDR) adjustment. Differential abundance of bacterial genera in UBD groups compared to the control was analyzed using DESeq2 with p.adj < 0.05, log2FC ≥ 1 and counts ≥50. Unclassified genera were excluded. Pairwise Wilcoxon rank sum test with FDR adjustment was performed on normalized counts on the genera determined by DESeq2. Different letters denote significant differences at p.adj < 0.05 between groups.

## Results

### Caloric intake and growth pattern

First, the effect of the UBD on intake and growth was determined. Total energy consumed by rats of the control group during the experiment was 40% higher than that by rats of the UBD group (Figure 1A). Accordingly, the UBD-fed rats gained significantly less weight and were significantly shorter compared to controls (Figures 1B,C). The length of the femur and lumbar (L3–L5) vertebrae—as measured by microCT, was also significantly shorter in rats from the UBD group (Figures 1D,E), demonstrating growth inhibition in the UBD-fed rats.

**Figure 2:**
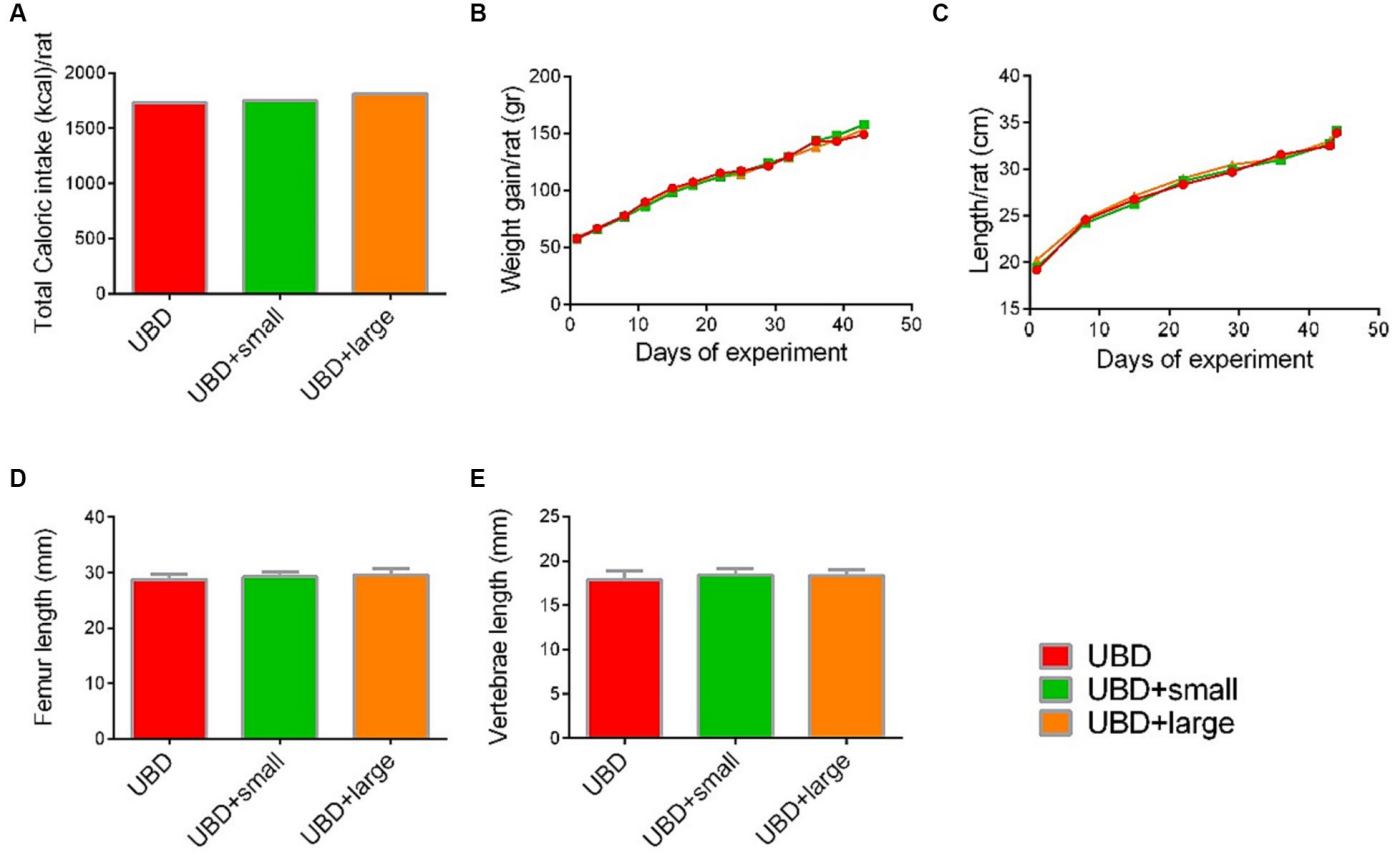
Effect of MFG supplementation on growth pattern and caloric intake during the experiment. **(A)** Total caloric intake (kcal/rat). **(B)** Weight gain (g/rat). **(C)** Longitudinal growth (cm/rat). **(D)** Femur length (mm). **(E)** Vertebrae length (mm). **(A)** Values are expressed as mean of 2 cages for each diet and calculated for the estimated consumption for rat. **(B–E)** Values are expressed as mean ± SD of *n* = 8 rats/group. Different superscript letters indicate significant difference (*p* < 0.05) by one-way ANOVA followed by Tukey’s test.

Next, we evaluated the effect of MFG gavage on caloric intake and growth. Total caloric intake, body weight and length, and length of the femora and L3–L5 vertebrae did not differ between the MFG-supplemented and non-supplemented UBD groups (Figures 2A–E). These results demonstrated that supplementation of small or large MFG has no beneficial effect on growth pattern.

To verify the cause for the differences in growth pattern throughout the experiment, we evaluated food utilization for weight gain. Caloric and macronutrient intakes were determined per cage. Energy and carbohydrate intakes were 28 and 26% lower in the UBD groups compared to the control group (Table 3). Protein consumption was almost 3-fold lower in rats of the UBD groups compared to controls, due to the diet composition as well as the lower food intake. Total intake of fat was comparable among all groups, due to the high content of fat in the UBD (50% higher than the recommended level).

**Table 3:** Nutrients consumed during the experiment.

Macronutrients	Control	UBD	UBD + small MFG	UBD + large MFG
Energy (kcal)	2417.5	1732.5	1689.8	1711.6
Carbohydrates (g)	384.3	281.5	273.8	276.9
Protein (g)	123.9	43.3	42.1	42.7
Fat (g)	42.7	48.1	47.3	48.1

Values are expressed as mean of 2 cages for each diet and calculated for the estimated consumption for rat for the whole experiment.

To determine the overall addition of fat, carbohydrates and proteins administrated by the MFG supplement was measured. End of sentences is missing Fat from MFG supplement added only 0.06 and 0.088% to the total fat consumption for the large and small MFG, respectively. The portion of protein and carbohydrate received through the MFG supplement were 0.31% and 0.35% for the small and 0.03% and 0.035% for the large MFG supplement, respectively.

**Figure 3:**
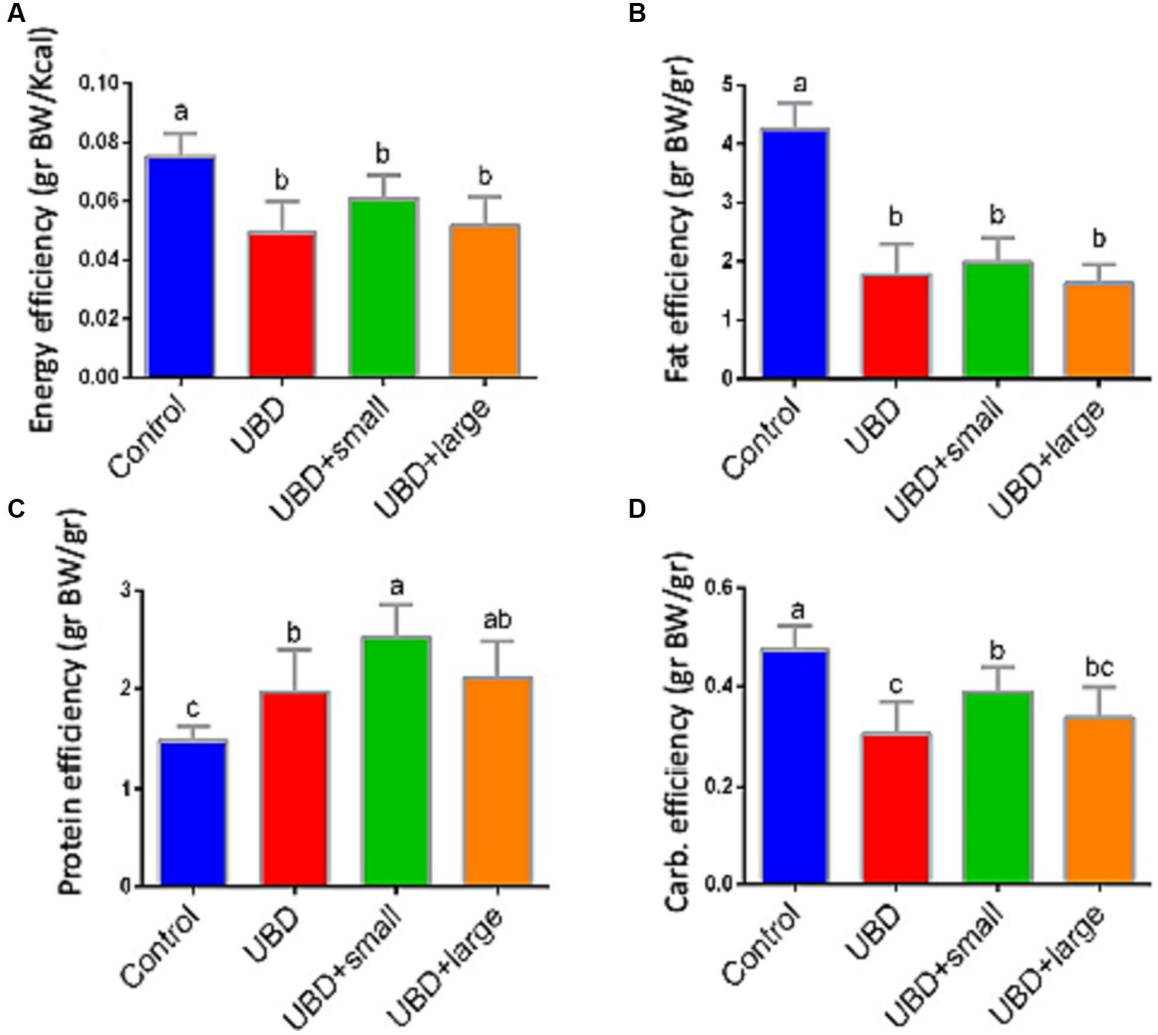
Food efficiency for weight gain. **(A)** Energy efficiency (g BW/Kcal). **(B)** Fat efficiency (g BW/g). **(C)** Protein efficiency (g BW/g). **(D)** Carbohydrate efficiency (g BW/g). Values are expressed as mean ± SD of *n* = 8 rats/group for control, UBD and UBD + small MFG, and *n* = 7 for UBD + large MFG. Different superscript letters indicate significant difference (*p* < 0.05) by one-way ANOVA followed by Tukey’s test.

Food utilization for weight gain was calculated individually for each rat considering the individual weight gain and the mean group consumption of each macronutrient. Energy and fat efficiencies for growth were significantly lower in the UBD groups compared to the control group (Figures 3A,B). Interestingly, protein efficiency was significantly higher in all UBD groups compared to the control group (Figure 3C), and significantly higher in the small MFG-supplemented group as compared to the non-supplemented UBD group. Carbohydrate efficiency was lower in all of the UBD groups compared to the control group (Figure 3D), but small MFG supplementation increased the efficiency compared to the non-supplemented UBD group. These results showed that supplementation of the UBD with small, but not large MFG enhances the efficiency of protein and carbohydrate utilization for weight gain.

### Effect of the unbalanced diet on bone morphology and mechanical properties

**Figure 4:**
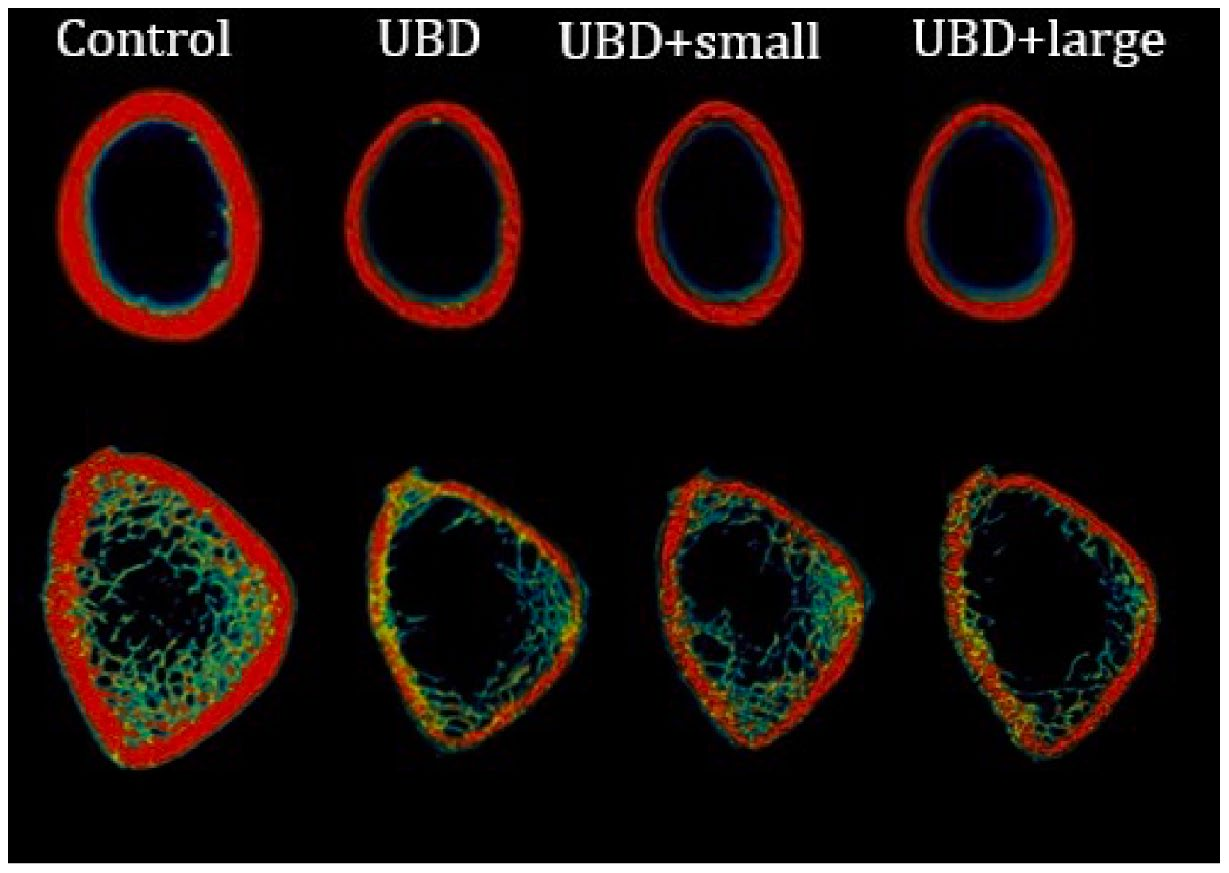
3D representation of the rats’ cortical (upper) and trabecular (lower) bone.

To explore the effects of the UBD and MFG supplementation on the skeletal system, microarchitecture examination of the femur and L3–L5 lumbar vertebrae was performed by MicroCT, to obtain cortical and trabecular bone parameters (Figure 4; Table 4).

**Table 4:** Morphometric and mechanical properties of femur and vertebral column.

The tested parameter				
**Femur cortical analysis**	**Control**	**UBD**	**UBD + small**	**UBD + large**
T.Ar (mm^2^)	11.3^a^ (1.08)	9.09^b^ (0.8)	9.8^b^ (0.75)	9.49^b^ (0.38)
B.Ar (mm^2^)	5.07^a^ (0.36)	3.53^b^ (0.37)	4.01^b^ (0.36)	3.56^b^ (0.37)
Ct.Ar/T.Ar (%)	44.94^a^ (1.93)	38.82^bc^ (1.85)	40.99^b^ (2.75)	37.54^c^ (2.35)
M.Ar (mm^2^)	6.24^a^ (0.77)	5.56^a^ (0.49)	5.79^a^ (0.57)	5.93^a^ (0.57)
Ct.Th (mm)	0.43^a^ (0.02)	0.33^bc^ (0.02)	0.36^b^ (0.03)	0.33^c^ (0.03)
BMD (g/cm^3^)	0.9^a^ (0.02)	0.89^a^ (0.02)	0.86^a^ (0.02)	0.9^a^ (0.1)
**Femur trabecular analysis**	**Control**	**UBD**	**UBD + small**	**UBD + large**
BV/TV %	27.74^a^ (3.31)	10.72^c^ (3.31)	16.92^b^ (4.22)	11.83^c^ (2.04)
Tb.Th (mm)	0.074^a^ (0.004)	0.072^ab^ (0.002)	0.07^b^ (0.002)	0.073^ab^ (0.003)
Tb.Sp (mm)	0.28^b^ (0.04)	0.71^a^ (0.11)	0.61^a^ (0.12)	0.71^a^ (0.12)
Tb.N (1/mm)	3.73^a^ (0.43)	1.46^c^ (0.43)	2.41^b^ (0.62)	1.62^c^ (0.26)
**Vertebral column L3-L5 trabecular analysis**	**Control**	**UBD**	**UBD + small**	**UBD + large**
BV/TV %	51.98 (6.7)	51.20 (8.2)	44.26 (6.3)	44.06 (5.1)
Tb.Th (mm)	0.19 (0.01)	0.20 (0.02)	0.18 (0.02)	0.18 (0.01)
Tb.Sp (mm)	0.27^b^ (0.03)	0.33^a^ (0.05)	0.34^a^ (0.02)	0.33^a^ (0.02)
Tb.N (1/mm)	2.70^a^ (0.16)	2.50^b^ (0.15)	2.39^b^ (0.11)	2.44^b^ (0.15)
**Three point bending test**	**Control**	**UBD**	**UBD + small**	**UBD + large**
Stiffness (*N*/mm)	266.33^a^ (39.5)	201.44^b^ (30.5)	237.03^ab^ (42.7)	217.7^ab^ (21.3)
Yield (*N*)	44.5^a^ (2.8)	27.54^b^ (8.07)	31.37^b^ (4.1)	29.47^b^ (5.6)
Fracture load (*N*)	79.09^a^ (18.2)	38.75^b^ (11.08)	37.1^b^ (10.2)	38.9^b^ (6.6)
Max load (*N*)	94.28^a^ (8.14)	62.9^b^ (8.1)	66.14^b^ (10.1)	63.6^b^ (7.8)
Energy to fracture (Nmm)	71.68^a^ (20.4)	64.2^a^ (13.5)	67.75^a^ (22.2)	65.87^a^ (23.37)

Bones were scanned by Micro-CT for determination of geometric parameters and BMD. After reconstruction, 2D and 3D analyses were performed. Cortical bone parameters: T.Ar, mean total area; B.Ar, mean bone area; Ct.Ar/Tt.Ar, cortical area fraction; Ma.Ar, medullary area; Ct.Th, cortical thickness; BMD, bone mineral density. Trabecular bone parameters: BV/TV, bone volume overtotal volume; Tb.Th, trabecular thickness; Tb.Sp, trabecular separation; Tb.N, trabecular number. Mechanical properties were evaluated by three-point bending experiment performed on bones of all mice from the four groups. Biomechanical parameters obtained from load–displacement curve: whole-bone stiffness (N/mm), yield point (N), fracture load (N), maximal load (N) and energy to fracture (N/mm). Values are expressed as mean ± SD of *n* = 8 rats/group for control, UBD and UBD + small MFG, and *n* = 7 for UBD + large MFG. Different superscript letters indicate significant difference (*p* < 0.05) by one-way ANOVA followed by Tukey’s test.

The UBD had significant negative effects on all the cortical bone parameters (Table 4), except for the medullary area. Significant improvements in cortical area fraction (mean bone area/mean total area) and cortical thickness were detected in the rats consuming UBD supplemented with small MFG compared to rats consuming UBD and UBD supplemented with large MFG. Surprisingly, despite the lower amounts of minerals in the UBD, BMD did not differ among the experimental groups. Trabecular bone is composed of a honeycomb-like network of trabecular plates and rods dispersed in the bone marrow compartment (9, 47). The comparison between the groups demonstrated significant differences in femur trabecular bone parameters. The percent bone volume (bone volume/total volume) and trabecular number decreased in the UBD groups, and the trabecular separation increased in these rats. Interestingly, rats receiving UBD supplemented with small MFG but not large MFG showed a significant improvement in these parameters (Figure 4). However trabecular parameters obtained from the L3–L5 vertebrae showed only marginal differences (Table 4). This could be attributed to the dissimilar mechanical loads applied on these parts of the skeleton.

To study the mechanical properties of the bone, we used a three-point bending experiment (Table 4). All the mechanical properties of the bones from rats fed the UBD showed deterioration compared to the control rats. MFG supplementation did not affect these properties.

### Fatty acid composition

The main FA for both MFG sizes used as a supplement to the UBD were myristic (C14:0), palmitic (C16:0), stearic (C18:0), and oleic (C18:1) acids (Table 5). These are the main FA found in milk. The concentrations of docosatetraenoic acid (C22:4 n6) and docosahexaenoic acid (DHA, C22:6 n3) were 10- and 30-fold higher in the small compared to large MFG, respectively (Table 6). These differences affected the total PUFA and total n-3 PUFA amounts, which were higher in the small MFG. As a consequence, the final ratio between omega-6 (n-6) PUFA and omega-3 (n-3) PUFA was 2:1 for small MFG, and 9:1 for large MFG (Table 6).

**Table 5:** FA composition in MFG.

FA acid	Small MFG	Large MFG
**SFA**		
C8:0 Caprylic acid	4.08	2.98
C10:0 Capric acid	7.37	7.35
C12:0 Lauric acid	6.46	7.46
C13:0 Tridecylic acid	0.14	0.19
C14:0 Mystric acid	15.99	17.04
C15:0 Pentadecylic acid	1.22	1.33
C16:0 Palmitic acid	30.70	32.80
C17:0 Margaric acid	0.44	0.44
C18:0 Stearic acid	7.33	7.16
C20:0 Archidic acid	0.06	0.06
**MUFA**		
C14:1 Myristoleic acid	1.08	1.08
C15:1 Cis-10-Pentadecenoic acid	0.24	0.26
C16:1n7 Palmitoleic acid	1.35	1.43
C17:1 cis-10-Heptadecenoic acid	0.17	0.20
C18:1n9 Elaidic acid	0.99	1.05
C18:1n9 oleic acid	16.30	15.10
C18:1n7 cis-Vaccenic acid	0.59	0.44
20:1n9 Gondoic acid	0.06	0.07
**PUFA**		
C18:2n6 Linolelaidic acid	0.35	0.32
C18:2n6 Linoleic acid	2.94	2.61
C18:3n6 γ-Linolenic acid	0.06	0.05
C18:3n3 α-Linolenic acid	0.31	0.30
C20:4n6 Arachidonic acid	0.10	0.10
C22:4n6 Docosatetraenoic acid	0.22	0.03
C22:6n3 Docosahexaenoic acid	1.36	0.05
Total SFA	73.79	76.82
Total MUFA	20.78	19.63
Total PUFA	5.34	3.45
Total n-6 PUFA	3.67	3.11
Total n-3 PUFA	1.67	0.35
n-6 to n-3 ratio	2.20:1	8.98:1

Values are in mol%.

**Table 6:** FA composition in the diets.

FA	Control	UBD	UBD + small	UBD + large
**SFA**				
C8:0 Caprylic acid	0.1	0.11	0.16	0.14
C10:0 Capric acid	0.02	0.01	0.12	0.1
C12:0 Lauric acid	0.02	0.12	0.23	0.23
C14:0 Mystric acid	0.74	0.55	0.84	0.81
C15:0 Pentadecanoic acid	0	0	0.02	0.02
C16:0 Palmitic acid	10.84	11	11.42	11.4
C17:0 Heptadecenoic acid	0	0	0.01	0.01
C18:0 Stearic acid	5.28	3.73	3.83	3.81
C20:0 Archidic acid	0.3	0.3	0.29	0.3
c23:0 Tricosylic acid	0.03	0.02	0.02	0.02
**MUFA**				
C14:1 Myristoleic acid	0	0	0.02	0.02
C16:1n7 Palmitoleic acid	0.25	0.36	0.38	0.38
C18:1n9 Elaidic acid	0.17	0.08	0.1	0.1
C18:1n9 Oleic acid	28.72	26.42	26.27	26.27
C18:1n7 cis-Vaccenic acid	2.23	1.97	1.94	1.94
20:1n9 Gondoic acid	0.35	0.33	0.32	0.32
C22:1cis13 Erucic acid	0.11	0.04	0.04	0.04
C24:1 Nervonic acid	0.06	0.05	0.05	0.05
**PUFA**				
C18:2n6 Linolelaidic acid	0.11	0.07	0.08	0.08
C18:2n6 Linoleic acid	45.05	48.47	46.56	46.69
C18:3n6 γ-Linolenic acid	0.31	0.04	0.04	0.04
C18:3n3 α-Linolenic acid	5.11	5.67	5.56	5.58
C20:4n6 Arachidonic acid	0.02	0.02	0.02	0.02
C20:5n3 Eicosapentaenoic acid	0.01	0.33	0.32	0.32
C22:2n6 Docosadienoic acid	0.05	0.01	0.01	0.01
C22:4n6 Docosatetraenoic acid	0	0.03	0.04	0.03
C22:6n3 Docosahexanoic acid	0.1	0.26	0.29	0.26
Total SFA	17.34	15.86	16.95	16.84
Total MUFA	31.89	29.24	29.12	29.12
Total PUFA	50.77	54.91	53.93	54.04
Total n-6 PUFA	45.55	48.65	47.76	47.88
Total n-3 PUFA	5.22	6.26	6.17	6.16
n-6 to n-3 ratio	8.73:1	7.78:1	7.74:1	7.78:1
Total fat consumption (g)	42.7	48.1	47.3	48.1

Values are in %. In figures “UBD + small MFG” is just “UBD + small,” same for large.

**Figure 5:**
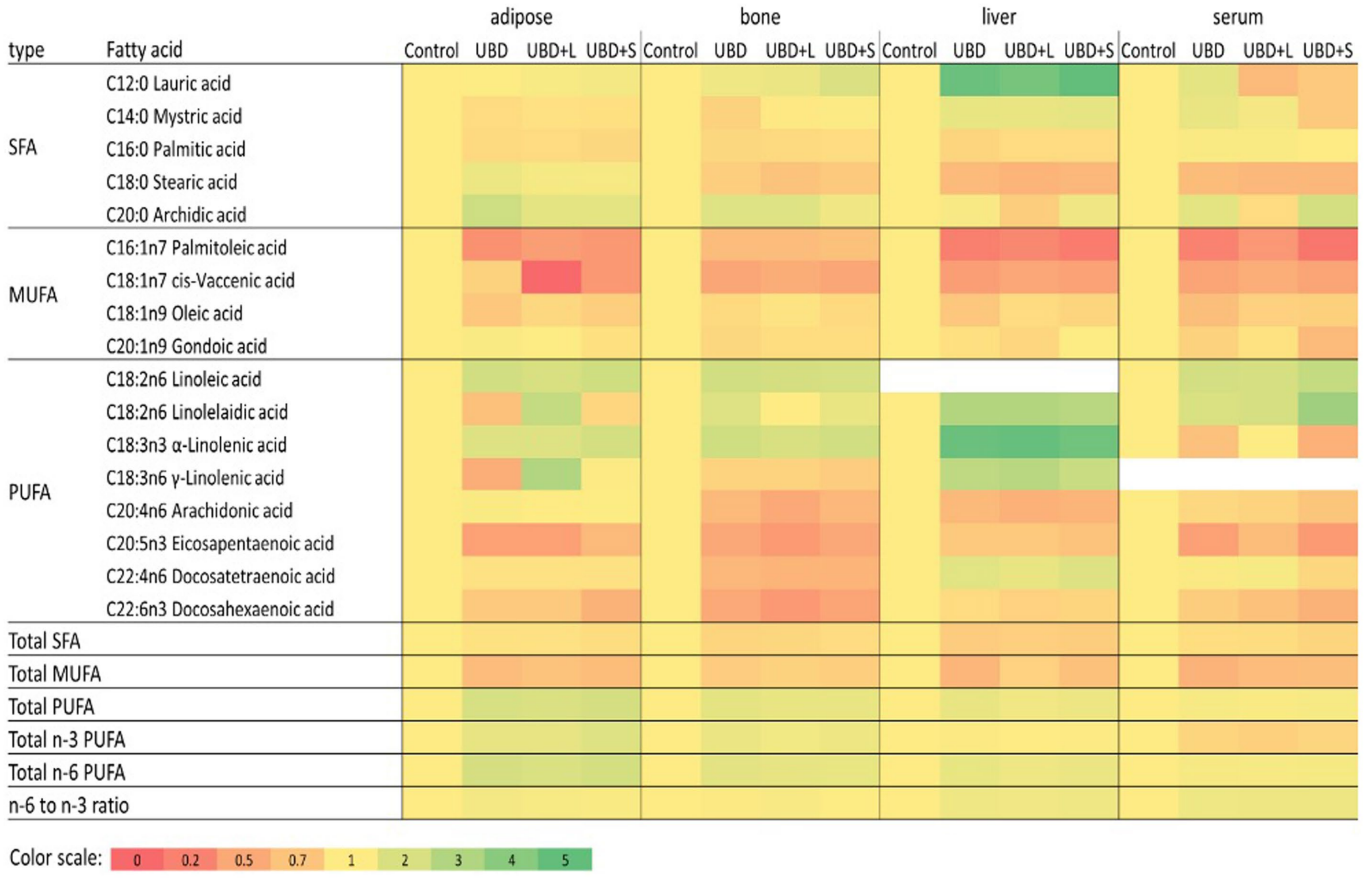
Relative fatty acid composition in adipose, bone, liver and serum. For each fatty acid and tissue data values of all treatments were divided with the relevant control value. Data relative to control is presented in a color heatmap with the color scale given below. Fatty acids that were detected in less than three of the four tissues were omitted from this figure. Detailed composition of the tissues is given in Supplemented data file (Supplementary Tables 1A–D). SFA, saturated fatty acids; MUFA, monounsaturated fatty acids.

FA composition of serum, liver, adipose tissue, and tibial bone marrow were determined at the end of the experiment. The enrichment or depletion in specific fatty acids in liver, bone marrow, adipose and serum compared with the control composition are presented in Figure 5. The composition of fatty acids in each of the above mentioned tissues are presented in Supplementary Tables 1A–D. The FA composition of the diets was calculated together with the FA composition of the MFG to assess the effect of the final diet on the rats’ tissues. The main FA found in the diets were palmitic (C16:0), stearic (C18:0), oleic (C18:1 n9), linoleic (C18:2 n6), and α-linolenic (C18:3n3) acids (Table 6). These are the main FA found in soybean oil, the main source of oil in the diets. Because the supplemented MFG introduced only a fraction of the total fat content in the diets, they had only a marginal effect on the diet’s FA composition. Nonetheless, odd-chain FA such as pentadecanoic (C15:0) and heptadecanoic (C17:0) acids originate from ruminants as a consequence of the rumen fermentation process and accordingly, these FA were found only in the MFG-supplemented groups.

The main FA in the serum and liver were palmitic (C16:0), stearic (C18:0), oleic (C18:1 n9), linoleic (C18:2 n6) and arachidonic (C20:4 n6) acids (Supplementary Tables 1A,B, respectively). In both tissues, UBD groups had lower concentrations of saturated FA (SFA) and monounsaturated FA (MUFA) and an increase in the percentage of PUFA compared to the control group (Supplementary Tables 1A,B, respectively).

The serum concentration of specific SFA, such as myristic (C14:0) and palmitic (C16:0) acids, did not differ between the small MFG-supplemented group and the control. Stearic acid (C18:0) decreased in the UBD groups compared to the control group. Arachidonic acid (C20:4 n6) did not differ between UBD, UBD + large MFG and the control groups. However, it was significantly higher in the UBD + small MFG group than in the control, with no difference between UBD + small MFG and non-supplemented UBD groups. For MUFA, palmitoleic (C16:1 n7) and oleic (C18:1) acids were lower in the UBD groups than the control group. For PUFA, linoleic (C18:2 n6) and α-linolenic (C18:3 n3) acids were significantly higher in the UBD groups than in the control group, concomitant with their higher concentrations in the UBD. Arachidonic acid and DHA (C22:6 n3) concentrations were lower in all the UBD groups compared to the control group, but in the UBD + small MFG group, they were significantly higher than in the non-supplemented UBD group (Supplementary Table 1D). The ratio between n-6 PUFA and n-3 PUFA was significantly higher in all the UBD groups compared to the control group (Supplementary Table 1A).

Most of the FA in the liver showed the same pattern as in the serum. For SFA, the UBD groups had significantly decreased palmitic (C16:0) and stearic (C18:0) acids compared to the control. For MUFA, palmitoleic (C16:1 n7) and cis-vaccenic (C18:1 n7) acids were also lower in the UBD groups. Oleic acid (C18:1 n9) was lower in the UBD group compared to the control. UBD + small MFG and UBD + large MFG groups did not differ from either the control or the non-supplemented UBD group. For PUFA, linoleic (C18:2 n6), γ-linolenic (C18:3 n6), eicosadienoic (C20:2 n6) and docosatetraenoic (C22:4 n6) acids were significantly higher in the UBD groups than in the control group. Arachidonic (C20:4 n6) and α-linolenic (C18:3 n3) acids were higher in the control group than in all the UBD groups. DHA (C22:6 n3) was higher in the control group than in the UBD + large MFG group. UBD and UBD + small MFG groups did not differ from either the control or the UBD + large MFG groups (Supplementary Table 1B). The ratio between n-6 PUFA and n-3 PUFA was significantly lower in the control group compared to all the UBD groups (Supplementary Table 1B).

In adipose tissue, the main FA were palmitic (C16:0), oleic (C18:1 n9), and linoleic (C18:2 n6) acids (Supplementary Table 1C). Unlike the liver and the serum, total SFA was only significantly higher in the control group compared to the UBD + small MFG group. Similar to the serum and liver, the total MUFA content was highest in the control group. In the UBD groups, total MUFA was significantly lower in the non-supplemented UBD and UBD + small MFG groups than in the UBD + large MFG group. Total PUFA was higher in all UBD groups compared to the control group (Supplementary Table 1C). For SFA, caprylic acid (C8:0) was lower in the non-supplemented UBD and UBD + large MFG groups compared to the control group. The UBD + small MFG group did not differ from any of the other groups. Lauric acid (C12:0) did not differ between the non-supplemented UBD group and the control. In the UBD + large MFG group, it was higher than in the control group. The UBD + small MFG group did not differ from any of the other groups. Palmitic acid was lower in all of the UBD groups compared to the control group. Arachidonic acid (C20:4 n6) was significantly higher in the non-supplemented UBD group than the control group. In the supplemented groups, arachidonic acid decreased compared to the UBD group, but was higher than the control group. For MUFA, the UBD groups showed a significant decrease in palmitoleic (C16:1 n7) and oleic acids, suggesting reduced metabolism in the adipose tissue. The UBD and UBD + small MFG groups had significantly lower amounts of oleic acid than the UBD + large MFG group. For PUFA, linoleic acid was significantly higher in the UBD + large MFG group than in all of the other groups. Linoleic acid increased in all the UBD groups, as did α-linolenic acid (C18:3 n3), the latter being significantly higher in the UBD + small MFG group than in the other groups (Supplementary Table 1C). The ratio between n-6 PUFA and n-3 PUFA was significantly lower in the control group compared to all UBD groups. The ratio was higher in the non-supplemented UBD group than in the UBD + small MFG group, and the ratio for the UBD + large MFG group did not differ from that of either the non-supplemented UBD or UBD + small MFG group (Supplementary Table 1C).

In the bone marrow, the major FA found were palmitic (C16:0), palmitoleic (C16:1 n7), oleic (C18:1 n9) and linoleic (C18:2 n6) acids (Supplementary Table 1D). As in the other tissues, all UBD groups showed a significant decrease in the percentage of SFA and increase in the proportion of PUFA compared to the control. However, supplementation of MFG decreased the percentage of PUFA compared to the non-supplemented UBD group, making the composition more similar to that of the control (Supplementary Table 1D). For the SFA, lauric acid (C12:0) did not differ between the non-supplemented UBD, UBD + large MFG and control groups. It was significantly lower in the UBD + small MFG group compared to the control, but did not differ from the non-supplemented UBD group. Myristic acid (C14:0) content also did not differ between the UBD groups and the control group. However, it was significantly higher in the UBD + small MFG vs. non-supplemented UBD group. Palmitic acid showed the same pattern as in the liver and the adipose tissue, i.e., a lower proportion in the UBD groups than in the control group. For MUFA, myristoleic acid (C14:1) was lower in the non-supplemented UBD and UBD + large MFG groups than in the control group. The UBD + small MFG group did not differ from the control. Palmitoleic acid decreased in all the UBD groups, similar to the liver, serum and adipose tissue. Oleic acid was lower in the non-supplemented UBD and UBD + small MFG groups than in the control group. The UBD + large MFG group did not differ from any of the other groups. For PUFA, there was an increase in linoleic acid in all the UBD groups compared to the control, similar to the other tissues. α-Linolenic (C18:3 n3) acid was significantly higher in the UBD groups than in the control group, but in the UBD + large MFG group, it was significantly lower than in the non-supplemented UBD group. Eicosapentaenoic acid (C20:5 n3) was lower in all of the UBD groups compared to controls (Supplementary Table 1D). Similar to the adipose tissue, the ratio between n-6 PUFA and n-3 PUFA was higher in all of the UBD groups except UBD + small MFG, which did not differ significantly from the control group (Supplementary Table 1D).

### Effect of unbalanced diet and MFG supplementation on gut microbiota

To explore the possible connection between MFG supplementation, the microbiome and bone parameters, we analyzed fecal samples obtained from the cecum of rats of all four dietary groups for microbiome composition and diversity. We conducted 16S sequencing of the V4 region with coverage of ~38,500 reads per sample.

First, we compared the distribution of the bacterial taxa in the groups after 6 weeks on their respective diets. An overview of the bacterial taxa is provided in Supplementary Figure S1A, showing the distribution of bacteria across the different phyla. The abundance of Actinobacteria was below 1% in all samples from each group (data not shown). We found an elevated abundance of Bacteroidetes and a lower abundance of Firmicutes in UBD + large MFG compared to the other groups. Furthermore, all UBD groups demonstrated a low abundance of Deferribacteres, which did not pass the minimal threshold in the control samples. The Deferribacteres phylum contained only the genus *Mucispirillum*. Finally, Verrucomicobria abundance was above 1% in several but not all samples of each group.

**Figure 6:**
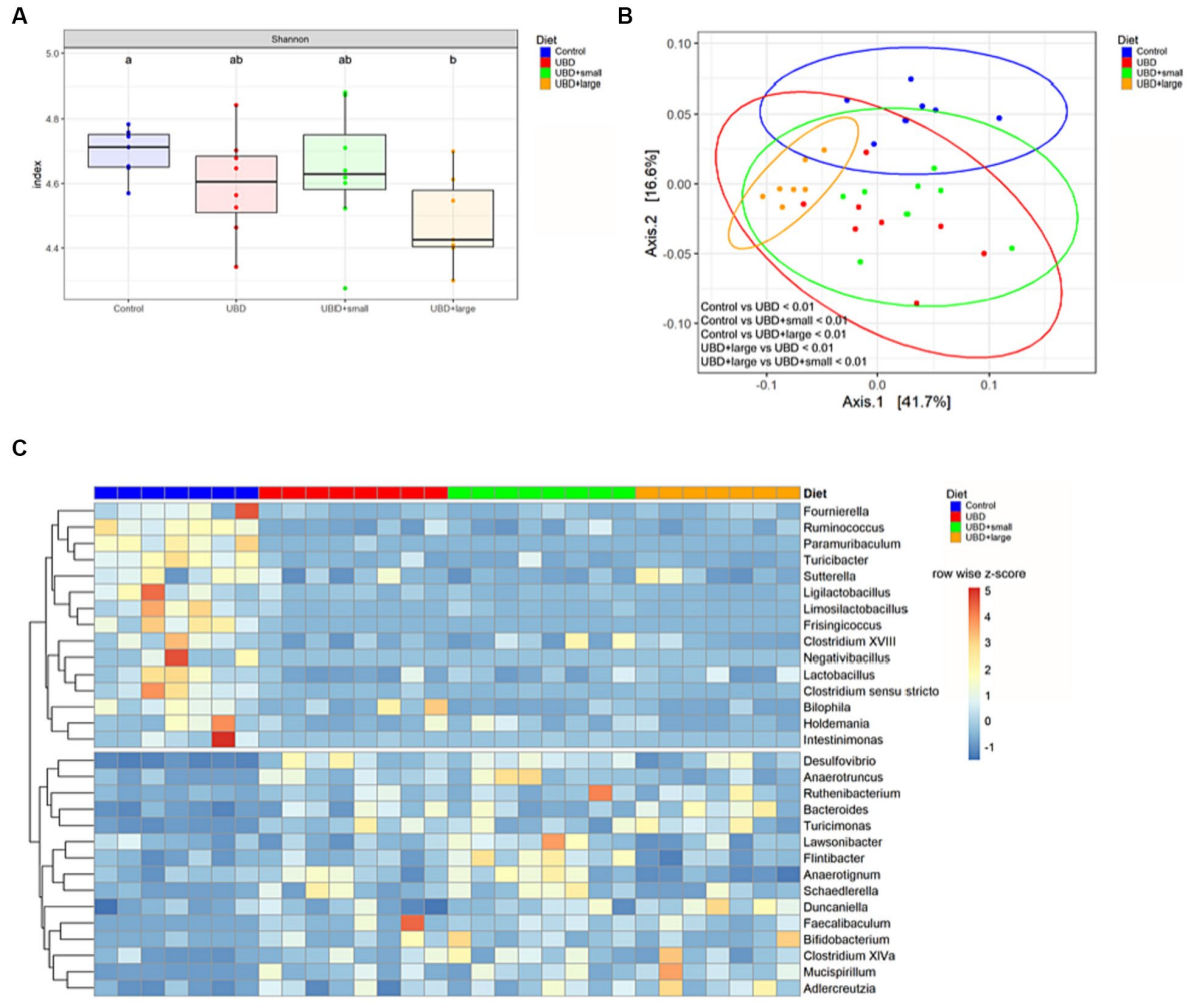
MFG supplementation does not correct UBD induced changes of the microbiome. **(A)** Alpha diversity was calculated using the Shannon index and a pairwise Wilcoxon rank sum test with FDR adjustment. Different letters denote significant differences at p.adj < 0.05 between groups. **(B)** PCoA plot based on weighted UniFrac distance. Plot ellipses represent the 95% confidence regions for group clusters. After testing for homogeneity of dispersion, subsequent PERMANOVA showed significant differences between the control and all UDB groups, as well as between UBD + large and the other UBD groups. *p* values were adjusted using Holm’s method. **(C)** Differential abundance of bacterial genera in UBD groups compared to the control, analyzed using DESeq2 with p.adj < 0.05, log2FC ≥ 1 and counts ≥50. Unclassified genera were excluded. Colors indicate row-wise z-score of relative genera abundance. *n* = 7 rats/group for control and UBD + large MFG, and *n* = 8 rats/group for UBD and UBD + small MFG.

The alpha diversity of all groups was measured to analyze the effects of UBD consumption with or without MFG. Only UBD + large MFG demonstrated a significant decrease in the Shannon index compared to the control (*p* = 0.042; Figure 6).

Subsequently, we analyzed the beta diversity based on the weighted UniFrac distance. The PCoA plot shows that samples from the control group and the UBD + large MFG group cluster mostly among themselves but not with the other UBD groups. In contrast, the UBD and UBD + small MFG samples show overlapping clusters. Performing PERMANOVA, the community composition was found to significantly differ between all groups (p.adj < 0.01) except for UBD vs. UBD + small MFG (p.adj = 0.393; Figure 6).

To determine bacterial genera that may mediate the effect of UBD consumption on the skeleton, differential abundance analysis was performed by comparing each UBD group with the control (Figure 1C). We identified 15 bacteria that were significantly less abundant in at least one of the UBD groups compared to the control. Of these, *Clostridium sensu stricto*, *Fournierella*, *Frisingicoccus*, *Intestinimonas*, *Ligilactobacillus*, *Limosilactobacillus*, *Paramuribaculum*, *Ruminococcus*, and *Turicibacter* were less abundant in all three UDB groups compared to the control (Supplementary Figure S1B) *Lactobacillus* and *Negativibacillus* were less abundant in the non-supplemented UBD and UBD + small MFG compared to the control, whereas *Clostridium* XVIII and *Sutterella* abundance was reduced only in UBD + small MFG compared to the control. *Bilophila* was less abundant in UBD + small MFG and UBD + large MFG compared to the control. *Holdemania* was less abundant in UBD + large MFG compared to the control. All these bacteria, except for *Paramuribaculum*, *Sutterella*, and *Bilophila*, belonged to the phylum Firmicutes.

Fifteen genera demonstrated significantly higher abundance in at least one UBD group compared to the control. *Bacteroides*, *Desulfovibrio*, *Faecalibaculum*, *Mucispirillum*, *Ruthenibacterium* and *Turicimonas* were more abundant in all three UBD groups compared to the control. *Anaerotruncus* and *Schaedlerella* were both higher in UBD and UBD + small MFG, whereas *Bifidobacterium*, *Clostridium* XlVa, *Flintibacter*, and *Lawsonibacter* were only significantly more abundant in UBD + small MFG compared to the control. *Adlercreutzia* was more abundant in UBD + small MFG and UBD + large MFG compared to the control. The UBD + large MFG also demonstrated a higher abundance of *Anaerotignum* and *Duncaniella* compared to the control. *Bacteroides* and *Duncaniella* belong to the phylum Bacteroidia, whereas *Mucispirillum* belongs to the phylum Deferribacteres. *Anaerotignum*, *Anaerotruncus*, *Clostridium* XlVa, *Faecalibaculum*, *Flintibacter*, *Lawsonibacter*, *Ruthenibacterium* and *Schaedlerella* are Firmicutes.

Subsequent analysis of differentially abundant microbes between non-supplemented UBD and UBD + small MFG or UBD + large MFG revealed that the *Bacteroidota* genus *Alistipes* was more abundant in UBD + large MFG compared to the non-supplemented UBD group, and no difference was detected compared to the control group (Supplementary Figure S1C). *Anaerotignum* abundance was significantly reduced in UBD + large MFG compared to all other groups, whereas *Flintibacter* abundance was significantly increased in UBD + small MFG compared to all other groups. *Ligilactobacillus* was less abundant in UBD + large MFG compared to the non-supplemented UBD group.

In summary, the UBD led to marked changes in the microbiome, whereas MFG supplementation had only a minimal effect, most pronounced in UDB + large MFG, despite UBD + small MFG exhibited the biggest bone parameter improvement compared to UBD without MFG supplementation.

## Discussion

The current study aimed to elucidate the effect of MFG on growth and development of postweaning rats fed a protein- and micronutrient-deficient diet, with a specific focus on bone quality and mechanical properties. The natural structure of the MFG was maintained to obtain new insights on the importance of its macrostructure and the relevance of globule size in guiding physiological processes. Previous studies have demonstrated the effect of isolated polar lipids on growth and metabolism, by using different streams of the dairy industry enriched with MFGM. However, the MFG’s natural macrostructure is destroyed during processing. Therefore, commercially available whey and butterfat fractions as a source for polar lipids and MFGM do not allow evaluating the effect of the natural structure of milk fat, or to distinguish between large and small MFG. Large and small MFG differ with respect to their lipidome and proteome and therefore, their bioactivities are expected to differ as well. The present study contributes to our understanding of the physiological role of MFG structure and size during early development.

In the current study, UBD consumption resulted in growth retardation and decreased weight gain. These results were common to all UBD groups, regardless of MFG supplementation. This result support our initial hypothesis that the protein and carbohydrate received as part of the MFG supplement have no or only marginal effect on the overall macronutrient consumed by the UBD groups. Moreover, the protein in the supplement is comparable to the protein in the bulk diet since both originate from cow milk and lactose in the supplement as a disaccharide is expected to have only marginal effect on top of the sucrose, a disaccharide used in the bulk diet. The reduction in growth rate of the UBD groups started early in the study, before reduced intake was recorded, suggesting that it was not a consequence of lower caloric intake. The fact that supplementation of small or large MFG did not change growth patterns compared to the UBD is in agreement with previous studies that found no effect on growth or weight gain in infants fed formula supplemented with MFGM (48, 49). Nonetheless, supplementation of MFGM as a complementary food for infants between 6 and 11 months of age improved weight gain, particularly in girls (50). These discrepancies highlight the need to understand the effect of structured lipids on growth trajectories and metabolism, which may differ at different stages of growth and age.

Although MFG supplementation did not affect caloric intake or growth in UBD fed rats, supplementation of small MFG to the UBD increased protein and carbohydrate utilization for growth. This means that less protein and carbohydrates were needed to support the same weight gain, and suggests differences in digestion and absorption efficiency. Small MFG contain more membrane than large MFG (51). Therefore, the UBD + small MFG had a greater MFGM content, which might explain the differences in protein utilization for growth because interactions between MFG and dietary proteins have been demonstrated—for example, a hydrophobic interaction between MFG and casein micelles in the stomach [reviewed by He et al. (52)]—and this may change protein digestion and absorption. Moreover, glycoproteins on the surface of the MFGM can reduce the rate of proteolysis by pepsin, thereby changing protein-digestion rates. Interestingly, we did not find any effect of MFG on fat utilization for growth, as might be expected based on previous studies in cows and broilers, where improved lipid digestion and absorption were found when polar lipids were supplemented to the diet (53, 54). This could be a result of the difference in the total fat content in the diets, which is typically much lower for these farm animals compared to the fat content in the UBD of the present study. Moreover, the very low dosage of MFG used in the present study also contributes to differences between the current results and previous *in vivo* studies.

All of the UBD had similar FA composition and PUFA content. In addition, the ratio between omega 6 and omega 3 was almost identical in the control and treatment groups. Although large and small MFG differ in their FA composition (16, 55), their contribution to the dietary FA composition was only marginal; the major source of dietary fat in the UBD groups was soy oil. Eating an UBD decreased the concentration of SFA and MUFA in all tissues compared to the control, excluding the SFA in the adipose tissue which decreased in the UBD + small MFG group. The reduction of MUFA, especially palmitoleic acid (c16;1), suggests a profound metabolic change in the UBD groups, usually associated with obesity [reviewed by Frigolet and Gutiérrez-Aguilar (56)].

MFG supplementation, especially small MFG, changed the FA composition in several tissues, making it more similar to that of the control. For example, in the bone marrow, supplementation of MFG reduced the level of PUFA compared to the non-supplemented UBD group. Moreover, in all examined tissues, the ratio between n-6 PUFA and n-3 PUFA was significantly higher in the UBD groups, although supplementation with small MFG reduced it, again making it more similar to the control. The ratio between omega 6 and omega 3 can influence the reactivity of lipid-mediated signaling molecules called eicosanoids (57). Depending on the initial substrates and the ratio between the substrates, different classes of eicosanoids are generated (58). Typically, eicosanoids formed from omega-6 precursors are considered proinflammatory (59, 60), whereas those from omega-3 PUFA are associated with reduced production of proinflammatory cytokines, specifically TNFα and interleukin (IL) 1 (61). Lau et al. (62) found that a lower ratio between n-6 and n-3 PUFA in the femur of young fat-1 transgenic mice correlated with stronger and healthier bones. In addition, an endogenous or exogenous source of omega-3 FA improved skeletal development and bone quality in these mice (44), and trabecular bone microarchitecture (43) and tibial BMD in young rats (63). In humans, Högström et al. (64) showed that omega-3 PUFA are positively associated with higher BMD in healthy young men. Thus, we assume that the ratio between n-6 PUFA and n-3 PUFA in small MFG contributed to better bone development. It should be noted that despite similar PUFA composition in the UBD, which was determined primarily by the oil used to formulate them, small MFG contain almost 5 times more n-3 PUFA than large MFG. This difference between small and large MFG can likely explain the differences in their effect on bone microarchitecture, which was improved when UBD was supplemented with small MFG. The fact that this was achieved without changing the overall composition of the diet supports our assumption that the structure of the dietary FA plays a role in metabolism and development.

## An unbalanced diet affects bone quality

The UBD used in the present study was low in protein and micronutrients.

Previous studies have shown that low protein intake changes endocrine signals that can affect bone mineralization, such as the concentration of parathyroid hormone (65) and insulin-like growth factor 1 (IGF-1) (66). In animal experiments, rats fed a low-protein diet presented inferior trabecular and cortical bone parameters compared to those fed a control diet (67–69). In terms of micronutrient deficiency, rats fed a diet with low concentrations of vitamin D, vitamin K, calcium, iron, magnesium, and phosphorus showed an adverse effect on bone microarchitecture (70–73). These changes in bone morphology are expected to alter its mechanical parameters. Concomitantly, we recorded lower values of stiffness, yield point, and maximum load in the UBD groups. However, energy to fracture was not altered by the treatment and was similar between groups. The UBD + small MFG group showed improvement in the microarchitecture of the bone compared to the other UBD groups, but this was not reflected in the mechanical tests. These results suggest that the improvement in bone microarchitecture was not big enough to be translated into bone strength.

### MFG supplementation does not modulate the effects of an UBD on the microbiome

One possible mechanism responsible for the differences in bone quality is a change in the gut microbiome. Several studies have linked dysbiosis to a variety of pathological conditions, negatively impacting the skeleton (74). Furthermore, MFGM harboring two forms of glycoconjugates (glycoproteins and glycolipids), are thought to have antimicrobial, anti-inflammatory, and prebiotic functions in the gut (75–78), thus changes in the composition and relative mass of the MFGM, represented in this study by UBD + small MFG and UBD + large MFG, could change the gut microbiome and consequently, bone growth and development.

In accordance with this study, the effects of unbalanced high-fat diets have been reported to influence microbiome composition in various organisms, mostly by altering the abundance of Bacteroidetes and Firmicutes (79–82). Our results showed significant differences in beta diversity and major changes in the abundance of various Firmicutes genera between all UDB groups and the control. However, the overall phylum abundance of Bacteroidetes and Firmicutes was only severely changed in UDB + large MFG compared to the control, which has also been reported for overweight high-fat diet-fed rats supplemented with additional polar lipid-enriched MFGM during pregnancy and lactation (83). A reduction in frequency of the Firmicutes phylum was reported in mice fed an ultraprocessed diet—which exhibited inferior bone parameters (45), as well as in patients with osteoporosis (84). Furthermore, only UBD + large MFG exhibited a reduced Shannon index and a diverging microbial composition compared to the other UBD groups. In total, our data indicates that the total abundance of Bacteroidetes and Firmicutes as well as alpha diversity do not necessarily correlate with bone quality, which was lower in all UBD groups compared to the control. The fact that changes in microbiome are not expressed in bone physical properties is like to be attributed to the deviation from the control microbiome composition. Microbial community composition may be affected by lipid utilization of large MFGs affects bacterial biofilm formation and growth (17). To further elucidate the effect of MFG on the microbiome bone axis, future studies should modify MFG supplementation in UBD groups to obtain a control like microbial composition.

Focusing on specific genera that could mitigate the effect of the UBD on the skeleton, the genus *Faecalibaculum* is positively correlated with IL1β (85), a downstream regulator of the effects of TNFα on inducing osteoclastogenesis and joint damage (86, 87). Furthermore, the abundance of several bacteria belonging to the class Clostridia, such as *Clostridium sensu stricto*, *Fournierella*, *Frisingicoccus*, *Intestinimonas*, *Ruminococcus*, and *Ruthenibacterium*, was either reduced or increased in all UBD groups compared to controls. Clostridia, and especially members of the family *Ruminococcaceae*, were strongly enriched after treatment of osteoporotic rats with parathyroid hormone (88), indicating one possible way in which the imbalance of Firmicutes observed in the UBD group could negatively impact bone development. Finally, the analysis excluded ASVs that could not be identified on the genus level and an involvement of these bacteria in bone development cannot be ruled out.

Neither small nor large MFG supplementation led to pronounced differences in the identified genera’s abundance between the UBD groups. From the four genera found to be differentially abundant in one of the MFG-supplemented groups compared to the non-supplemented UBD group, only two have been linked to bone remodeling: the species *Alistipes indistinctus* was found to be lower in senile osteoporotic rats (89) and its increased abundance in UBD + large MFG compared to controls highlights this genus as an interesting candidate for future research. Jung et al. (90) showed that spent culture supernatant of *Ligilactobacillus salivarius* strain MG2645 reduces osteoclastogenesis-related gene expression in RAW 264.7 macrophages, but the connection between *Ligilactobacillus* and MFG is unclear. Considering the similarity of non-supplemented UBD and UBD + large MFG bone phenotype, the even stronger reduction in *Ligilactobacillus* abundance may not be biologically relevant.

Because the dysbiosis following UBD consumption was not improved by MFG administration, the observed phenotypical bone improvements in UBD + small MFG are likely related to another mechanism that connects MFG with bone development.

### Synopsis: the importance of food structure

Rats were fed a formulated diet that mimics the Western diet—characterized by the consumption of ultraprocessed foods that are high in sugar and fat, and low in protein, fiber, and micronutrients. Ultraprocessed food is usually stripped of its natural structure and therefore, the importance of structure is rarely studied. Milk fat is secreted in a complex structure that is profoundly different from processed, isolated dietary ingredients and from plant derived dietary oils (91). Milk-fat composition is dominated by its triglyceride core (fat). However, the composition and content of the bioactive trilayer of polar lipids, glycoconjugates, and proteins enveloping the triglyceride droplet are altered by the size of the milk fat globule. Here, the natural structure of milk fat was studied in relation to the development and health of animals fed an unbalanced diet. To the best of our knowledge, this is the first study which aims to assign a physiological role to the size diversity of milk fat globules present in milk. The potential of milk fat, in its natural structure, to alleviate the detrimental effects of the unbalanced diet is demonstrated.

## Data availability statement

The original contributions presented in the study are publicly available. This data can be found at: https://www.ncbi.nlm.nih.gov/geo/ with the accession number: GSE239878.

## Ethics statement

The animal study was approved by the Hebrew University Ethics Committee AG-18-15578-3. The study was conducted in accordance with the local legislation and institutional requirements.

## Author contributions

NA-A: Conceptualization, Data curation, Formal analysis, Funding acquisition, Investigation, Methodology, Project administration, Resources, Software, Supervision, Validation, Visualization, Writing – original draft, Writing – review & editing. HA: Data curation, Formal analysis, Visualization, Writing – original draft. JJ: Data curation, Formal analysis, Writing – original draft. SD: Data curation, Visualization, Writing – original draft. CR: Methodology, Writing – review & editing, Writing – original draft. RM-S: Formal analysis, Writing – original draft. SP: Formal analysis, Writing – original draft. EM-O: Conceptualization, Data curation, Formal analysis, Funding acquisition, Investigation, Methodology, Resources, Supervision, Validation, Writing – original draft, Writing – review & editing.
