# Supplementary material for: Effect of milk fat globules on growth and metabolism in rats fed an unbalanced diet
Source: Front Nutr. 2024 Jan 11;10:1270171. doi: 10.3389/fnut.2023.1270171 (PMC10808575; doi:10.3389/fnut.2023.1270171)
Supplement: Supplementary file 1 [file Data_Sheet_1.PDF]

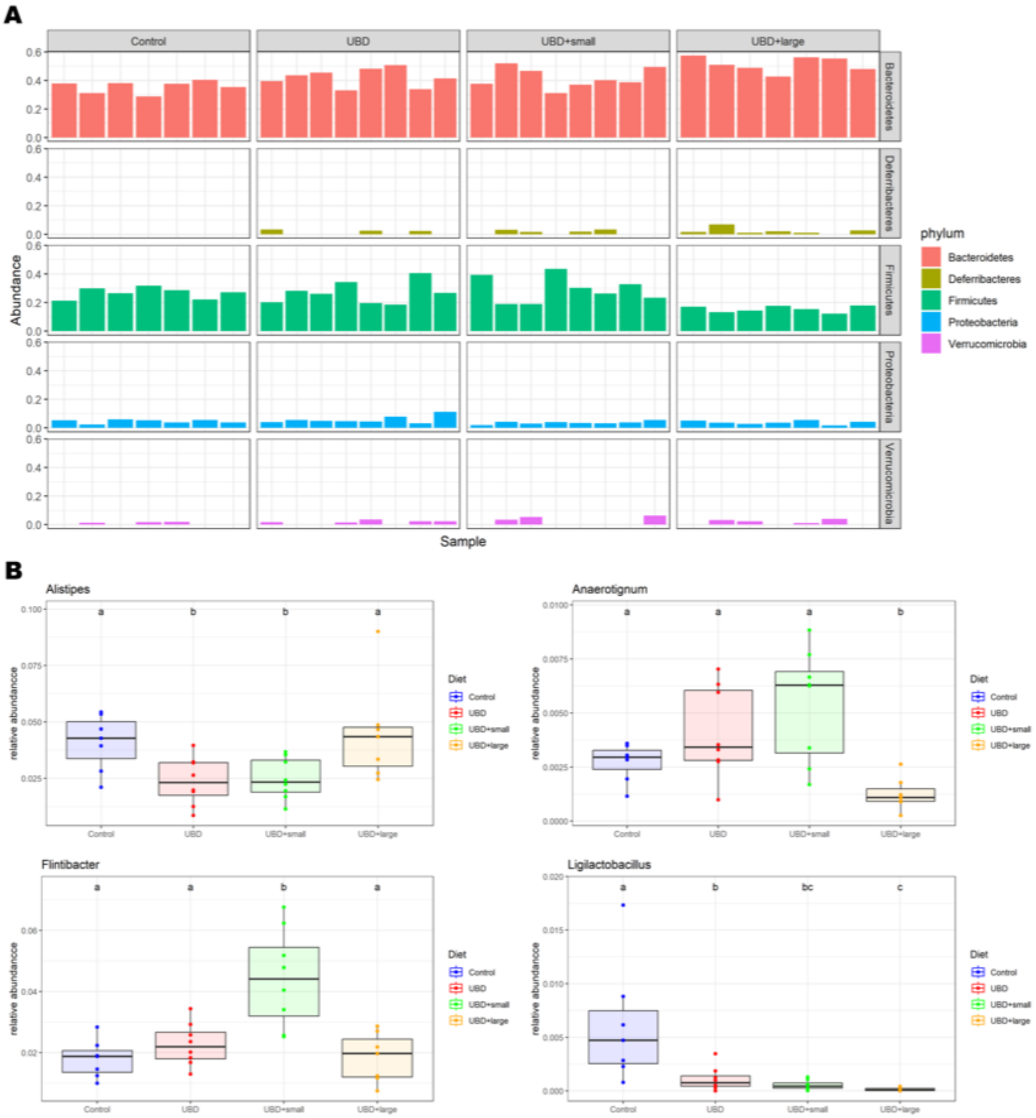

Figure S1. Phylum composition of the gut microbiota and differential abundant bacteria after MFG supplementation. **(A)** Distribution of the bacterial taxa at the phylum level per sample of each group. Only ASVs above the minimum frequency of 1% are included. **(B)** Differential abundance of bacterial genera in UBD+small and UBD+large groups compared to the UBD control, analyzed using DESeq2 with p.adj < 0.05, log2FC ≥ 1 and counts ≥ 50. Unclassified genera were excluded. Subsequent pairwise Wilcoxon rank sum test with FDR adjustment was performed on normalized counts of the genera. Different letters denote significant differences at p.adj < 0.05 between groups. n = 7 rats/group for control and UBD+large MFG, and n = 8 rats/group for UBD and UBD+small MFG.

Table 1A. FA composition in the serum. Values are in mol% (±SD).

FA	Control	UBD	UBD+small	UBD+large
SFA				
C12:0 Lauric acid	0.09^{a} (0.04)	0.15^{a} (0.1)	0.07^{a} (0.07)	0.06^{a} (0,04)
C14:0 Mystric acid	0.5^{b} (0.08)	0.77^{a} (0.27)	0.39^{b} (0.17)	0.64^{ab} (0.08)
C15:0 Pentadecanoic acid	0.24 (0.08)	0.25 (0.06)	0.24 (0.28)	0.28 (0.12)
C16:0 Palmitic acid	16.50^{b} (0.6)	18.67^{a} (0.7)	17.29^{b} (1.8)	19.10^{a} (0.85)
C18:0 Stearic acid	19.08^{a} (1.3)	12.96^{b} (0.97)	12.51^{b} (1.3)	12.50^{b} (1.0)
C20:0 Archidic acid	0.09^{b} (0.6)	0.15^{ab} (0.05)	0.19^{a} (0.06)	0.08^{b} (0.08)
MUFA				
C16:1n7 Palmitoleic acid	2.34^{a} (0.34)	0.69^{b} (0.18)	0.50^{b} (0.24)	1.01^{b} (0.05)
C18:1n9 Oleic acid	12.23^{a} (0.88)	8.51^{b} (0.97)	10.02^{b} (1.9)	10.08^{b} (2.1)
C18:1n7 cis-Vaccenic acid	2.5 (0.19)	1.3 (0.17)	1.29 (0.05)	1.46 (0.27)
C20:1n9 Gondoic acid	0.18^{a} (0.05)	0.15^{a} (0.05)	0.12^{a} (0.06)	0.17^{a} (0.12)
C24:1 Nervonic acid	0.33 (0.2)	0.14 (0.03)	0.23 (0.1)	0.28 (0.17)
PUFA				
C18:2n6 Linoleic acid	12.87^{c} (1.8)	27.50^{b} (1.2)	32.21^{a} (4.5)	26.42^{b} (2.3)
C18:3n6 γ-Linolenic acid	0.53^{a} (0.23)	0.38^{a} (0.11)	0.32^{a} (0.16)	0.56^{a} (0.26)
C18:3n3 α-Linolenic acid	0.39^{c} (0.1)	0.77^{b} (0.12)	1.34^{a} (0.55)	0.81^{b} (0.24)
C20:4n6 Arachidonic acid	27.67^{a} (1.6)	24.19^{b} (0.79)	20.58^{c} (3.8)	23.19^{bc} (2.3)
C20:5n3 Eicosapentaenoic acid	0.48 (0.9)	0.24 (0.04)	0.22 (0.1)	0.33 (0.17)
C22:4n6 Docosatetraenoic acid	0.29 (0.07)	0.32 (0.04)	0.25 (0.05)	0.35 (0.25)
C22:6n3 Docosahexaenoic acid	3.08^{a} (0.23)	2.46^{b} (0.17)	1.9^{c} (0.1)	2.23^{bc} (0.38)
Total SFA	35.98^{a} (1.8)	32.95^{b} (0.96)	30.69^{c} (1.1)	32.61^{bc} (0.96
Total MUFA	17.46^{a} (1.05)	10.78^{b} (1.2)	12.16^{b} (0.8)	11.96^{b} (1.8)
Total PUFA	46.56^{b} (2.2)	56.26^{a} (1.3)	57.14^{a} (1.0)	54.65^{a} (2.4)
Total n-6 PUFA	42.04^{b} (2.6)	52.39^{a} (1.2)	53.14^{a} (2.6)	50.95^{a} (1.17)
Total n-3 PUFA	4.52^{a} (0.39)	3.87^{b} (0.21)	3.81^{b} (0.2)	3.70^{b} (0.32)
n-6 to n-3 ratio	9.4^{b}:1 (1.2)	13.5^{a}:1 (0.76)	13.9^{a}:1 (0.8)	13.9^{a}:1 (1.8)

Table 1B. FA composition in the liver. Values are in mol%, mean (±SD).

FA	Control	UBD	UBD+small	UBD+large
SFA				
C12:0 Lauric acid	0.06^{a} (0.03)	0.29^{a} (0.19)	0.3^{a} (0.45)	0.27^{a} (0.29)
C14:0 Mystric acid	0.69^{a} (0.13)	1.1^{a} (0.45)	1.13^{a} (0.2)	1.08^{a} (0.22)
C16:0 Palmitic acid	22.71^{a} (2.2)	19.38^{b} (1.1)	20.36^{b} (1.4)	20.19^{b} (1.4)
C18:0 Stearic acid	21.06^{a} (2.1)	14.29^{b} (1.6)	13.82^{b} (2)	13.16^{b} (1.6)
C20:0 Archidic acid	0.05^{a} (0.03)	0.06^{a} (0.03)	0.07^{a} (0.01)	0.04^{a} (0.02)
C22:0 Docosanoic acid	0.07^{a} (0.03)	0.08^{a} (0.07)	0.06^{a} (0.04)	0.04^{a} (0.02)
MUFA				
C16:1n7 Palmitoleic acid	3.2^{a} (0.8)	0.84^{b} (0.29)	0.78^{b} (0.15)	1.05^{b} (0.43)
C18:1n9 Oleic acid	15.65^{a} (2.2)	11.8^{b} (1.7)	13.36^{ab} (1.2)	14.02^{ab} (1.4)
C18:1n7 cis-Vaccenic acid	3.34^{a} (0.24)	1.6^{b} (0.09)	1.7^{b} (0.14)	1.8^{b} (0.09)
C20:1n9 Gondoic acid	0.14^{a} (0.06)	0.13^{a} (0.03)	0.15^{a} (0.04)	0.12^{a} (0.03)
PUFA				
C18:2n6 Linoleic acid	10.05^{b} (1.2)	29.81^{a} (1.8)	28.48^{a} (2.7)	29.81^{a} (1.9)
C18:3n6 γ-Linolenic acid	0.25^{b} (0.05)	0.67^{a} (0.16)	0.59^{a} (0.13)	0.71^{a} (0.19)
C18:3n3 α-Linolenic acid	0.29^{b} (0.06)	1.4^{a} (0.22)	1.36^{a} (0.25)	1.43^{a} (0.2)
C20:2n6 Eicosadienoic acid	0.25^{b} (0.08)	0.48^{a} (0.04)	0.46^{a} (0.08)	0.48^{a} (0.08)
C20:4n6 Arachidonic acid	19.99^{a} (1.9)	13.33^{b} (1.5)	12.57^{b} (1.9)	12.1^{b} (1.6)
C20:5n3 Eicosapentaenoic acid	0.26^{a} (0.08)	0.2^{a} (0.05)	0.19^{a} (0.01)	0.2^{a} (0.03)
C22:4n6 Docosatetraenoic acid	0.26^{b} (0.03)	0.46^{a} (0.07)	0.48^{a} (0.3)	0.4^{a} (0.03)
C22:6n3 Docosahexaenoic acid	4.06^{a} (0.3)	3.59^{ab} (0.34)	3.47^{ab} (0.56)	3.35^{b} (0.59)
Total SFA	44.66^{a} (1)	35.22^{b} (1.9)	35.76^{b} (2.2)	36.33^{b} (1.4)
Total MUFA	22.33^{a} (3.1)	14.37^{b} (1.9)	15.97^{b} (1.3)	18.46^{b} (1.7)
Total PUFA	32.44^{b} (3.1)	49.97^{a} (1.3)	47.81^{a} (1.3)	44.76 ^{a} (1.2)
Total n-6 PUFA	27.63^{b} (2.8)	44.37^{a} (1.3)	42.36^{a} (1.3)	39.53^{a} (1.1)
Total n-3 PUFA	4.63^{b} (0.34)	5.2^{a} (0.22)	5.03^{ab} (0.42)	4.84^{ab} (0.41)
n-6 to n-3 ratio	5.9^{b}:1 (0.37)	8.5^{a}:1 (0.52)	8.4^{a}:1 (0.84)	8.1^{a}:1 (0.73)

Total n-6 PUFA and dependent values off

Table 1C. FA composition in the adipose tissue. Values are in mol% (±SD).

FA	Control	UBD	UBD+small	UBD+large
SFA				
C8:0 Caprylic acid	0.20[a] (0.05)	0.11[b] (0.05)	0.14[ab] (0.06)	0.08[b] (0.04)
C10:0 Capric acid	0.05[a] (0.02)	0.05[a] (0.01)	0.05[a] (0.01)	0.06[a] (0.03)
C12:0 Lauric acid	0.19[b] (0.01)	0.20[b] (0.03)	0.25[ab] (0.03)	0.23[a] (0.03)
C14:0 Mystric acid	2.07[a] (0.1)	1.84[a] (0.18)	1.92[a] (0.23)	1.93[a] (0.18)
C16:0 Palmitic acid	30.03[a] (1.0)	26.34[b] (1.8)	25.88[b] (1.8)	26.73[b] (1.4)
C18:0 Stearic acid	3.70 (0.25)	5.51 (0.93)	4.59 (0.75)	4.65 (0.82)
C20:0 Archidic acid	0.09[c] (0.03)	0.21[a] (0.04)	0.15[b] (0.03)	0.15[b] (0.03)
C24:0 Lignoceric acid	0.05 (0.03)	0.05 (0.05)	0.07 (0.03)	0.09 (0.06)
MUFA				
C16:1n7 Palmitoleic acid	10.44[a] (0.7)	4.12[b] (0.77)	4.47[b] (1.0)	4.95[b] (0.96)
C18:1n9 Oleic acid	32.84[a] (1.3)	24.83[c] (1.3)	26.4[c] (1.7)	28.79[b] (0.8)
C18:1n7 cis-Vaccenic acid	3.77 (0.61)	3.14 (1.48)	1.62 (1.6)	0.42 (0.09)
C20:1n9 Gondoic acid	0.14[a] (0.02)	0.16[a] (0.04)	0.13[a] (0.02)	0.15[a] (0.03)
PUFA				
C18:2n6 Linolelaidic acid	0.14[b] (0.07)	0.10[b] (0.07)	0.12[b] (0.08)	0.35[a] (0.05)
C18:2n6 Linoleic acid	13.7[b] (1.01)	29.87[a] (2.2)	30.39[a] (2.6)	27.71[a] (1.8)
C18:3n6 γ-Linolenic acid	0.12 (0.01)	0.07 (0.01)	0.13 (0.05)	0.36 (0.15)
C18:3n3 α-Linolenic acid	1.16[c] (0.09)	2.12[b] (0.19)	2.46[a] (0.3)	2.14[b] (0.23)
C20:4n6 Arachidonic acid	0.73[a] (0.09)	0.83[a] (0.25)	0.77[a] (0.18)	0.76[a] (0.43)
C20:5n3 Eicosapentaenoic acid	0.06 (0.02)	0.03 (0.01)	0.04 (0.01)	0.03 (0.01)
C22:4n6 Docosatetraenoic acid	0.15[a] (0.06)	0.14[a] (0.05)	0.14[a] (0.04)	0.14[a] (0.08)
C22:6n3 Docosahexaenoic acid	0.18[a] (0.07)	0.14[a] (0.06)	0.11[a] (0.05)	0.14[a] (0.07)
Total SFA	36.42[a] (1.3)	34.33[ab] (2.2)	33.06[b] (2.3)	33.94[ab] (1.4)
Total MUFA	47.2[a] (0.95)	32.27[c] (0.1)	32.64[c] (1.1)	34.32[b] (1.4)
Total PUFA	16.37[b] (0.1)	33.39[a] (2.4)	34.29[a] (2.7)	31.73[a] (2.2)
Total n-6 PUFA	14.87[b] (0.9)	31.02[a] (2.3)	31.57[a] (2.5)	29.34[a] (2.1)
Total n-3 PUFA	1.41 (0.07)	2.30 (0.22)	2.63 (0.28)	2.32 (0.24)
n-6 to n-3 ratio	10.5[c]:1 (0.6)	13.5[a]:1 (1.3)	12[b]:1 (0.86)	12.7[ab]:1 (1.1)

Table 1D. FA composition in the bone marrow. Values are in mol% (±SD).

FA	Control	UBD	UBD+small	UBD+large
SFA				
C10:0 Capric acid	0.04^{a} (0.03)	0.08^{a} (0.04)	0.08^{a} (0.04)	0.04^{a} (0.02)
C12:0 Lauric acid	0.28^{b} (0.28)	0.4^{ab} (0.12)	0.54^{a} (0.12)	0.43^{ab} (0.05)
C13:0 Tridecylic acid	0.05 (0.01)	0.04 (0.02)	0.05 (0.04)	0.04 (0.01)
C14:0 Mystric acid	2.51^{ab} (0.12)	2.09^{b} (0.2)	2.71^{a} (0.24)	2.46^{ab} (0.17)
C15:0 Pentadecanoic acid	0.27(0.05)	0.17 (0.01)	0.25 (0.08)	0.2 (0.02)
C16:0 Palmitic acid	24.08^{a} (1.7)	20.69^{b} (1.01)	21.7^{b} (0.88)	21.17^{b} (1.3)
C17:0 Heptadecenoic acid	0.17^{a} (0.02)	0.14^{a} (0.02)	0.14^{a} (0.01)	0.13^{a} (0.02)
C18:0 Stearic acid	6.45 (2.1)	5.21 ((0.77)	5.03 (0.65)	4.69 (0.74)
C20:0 Archidic acid	0.05^{a} (0.02)	0.09^{a} (0.03)	0.07^{a} (0.02)	0.09^{a} (0.08)
MUFA				
C14:1 Myristoleic acid	0.59^{a} (0.12)	0.38^{c} (0.05)	0.51^{ab} (0.09)	0.42^{bc} (0.08)
C15:1 Cis-10-Pentadecenoic acid	0.08 (0.07)	0.04 (0.01)	0.07 (0.04)	0.04 (0.01)
C16:1n7 Palmitoleic acid	13.6^{a} (2.4)	9.28^{b} (0.9)	9.88^{b} (1.5)	9.29^{b} (1.2)
C17:1 cis-10-Heptadecenoic acid	0.24 (0.05)	0.18 (0.05)	0.19 (0.01)	0.18 (0.03)
C18:1n9 Oleic acid	27.04^{a} (2.1)	23.86^{b} (0.97)	23.81^{b} (1.1)	25.62^{ab} (1.1)
C18:1n7 cis-Vaccenic acid	3.33 (0.67)	1.76 (0.37)	1.77 (0.2)	1.93 (0.35)
C20:1n9 Gondoic acid	0.22^{a} (0.1)	0.19^{a} (0.04)	0.2^{a} (0.06)	0.2^{a} (0.05)
PUFA				
C18:2n6 Linolelaidic acid	0.07 (0.03)	0.13 (0.13)	0.11 (0.03)	0.07 (0.01)
C18:2n6 Linoleic acid	13.01^{b} (0.8)	28.48^{a} (1.5)	26.45^{a} (1.2)	27.2^{a} (1.1)
C18:3n6 γ-Linolenic acid	0.19 (0.06)	0.16 (0.01)	0.15 (0.02)	0.16 (0.07)
C18:3n3 α-Linolenic acid	0.90^{c} (0.08)	1.99^{a} (0.14)	1.94^{ab} (0.15)	1.8^{b} (0.09)
C20:2n6 Eicosadienoic acid	0.46^{a} (0.16)	0.46^{a} (0.1)	0.4^{a} (0.07)	0.38^{a} (0.08)
C20:4n6 Arachidonic acid	4.47 (1.1)	2.99 (0.61)	2.89 (0.39)	2.51 (0.43)
C20:5n3 Eicosapentaenoic acid	0.11^{a} (0.02)	0.06^{b} (0.02)	0.06^{b} (0.02)	0.05^{b} (0.01)
C22:2n6 Docosadienoic acid	0.42 (0.36)	0.18 (0.18)	0.09 (0.05)	0.09 (0.07)
C22:4n6 Docosatetraenoic acid	0.54 (0.19)	0.35 (0.06)	0.34 (0.04)	0.34 (0.06)
C22:6n3 Docosahexanoic acid	0.52 (0.27)	0.29 (0.13)	0.27 (0.06)	0.23 (0.07)
Total SFA	33.92^{a} (3.2)	28.95^{b} (1.4)	30.61^{b} (1.4)	29.27^{b} (1.9)
Total MUFA	45.12 (4.3)	35.71 (1.1)	36.45 (1.9)	37.71 (1.7)
Total PUFA	20.94^{c} (1.5)	35.32^{a} (1.2)	32.92^{b} (1.3)	33.0^{b} (1.4)
Total n-6 PUFA	18.73^{c} (1.2)	32.31^{a} (1.1)	30.05^{b} (1.1)	30.4^{b} (1.3)
Total n-3 PUFA	1.54^{b} (0.2)	2.35^{a} (0.15)	2.28^{a} (0.18)	2.08^{a} (0.15)
n-6 to n-3 ratio	12.2^{c}:1 (1.2)	13.7^{ab}:1 (0.8)	13.1^{bc}:1 (0.6)	14.5^{a}:1 (0.7)
